# Children’s Play Profiles: Contributions From Child’s Temperament and Father’s Parenting Styles in a Portuguese Sample

**DOI:** 10.3389/fpsyg.2020.01978

**Published:** 2020-08-28

**Authors:** Carolina Santos, Lígia Monteiro, Olívia Ribeiro, Brian E. Vaughn

**Affiliations:** ^1^Centre for Social Research and Intervention, Iscte-Instituto Universitário de Lisboa, Lisbon, Portugal; ^2^William James Center for Research, ISPA-Instituto Universitário, Lisbon, Portugal; ^3^Human Development and Family Studies, Auburn University, Auburn, AL, United States

**Keywords:** play, social behaviors, non-social behaviors, temperament, father, preschool

## Abstract

Using a sample of Portuguese preschool-age children, we aimed to identify different play profiles based on teachers’ descriptions of social and non-social behaviors, as well as characterize them in terms of children’s characteristics (sex and temperament) and fathers’ parenting styles (e.g., warmth and involvement or punitive strategies). The 243 children were distributed across four profiles (identified through a two-stage cluster analysis): Solitary/Reticent, Social Rough, Social, and Social Solitary. A univariate effect was found between play profiles and children’s effortful control, as well as fathers’ punitive strategies. In addition, a significant multivariate interaction was found between child’s sex and the Solitary/Reticent and Social Rough profiles for father’s punitive strategies. In this sample, children with social play profiles seem to use diverse types of behaviors during their interactions with peers and in being adjusted within the group. As children’s early experiences with peers are a central context for healthy development, a better understating of the diversity of play profiles and its predictors is important for early interventions.

## Introduction

In the field of human development, there is consensus that peer interactions provide unique and essential opportunities for children’s socio-emotional, cognitive, and behavioral development (Coplan and Arbeau, 2009). In the context of these interactions, opportunities emerge, not only for practicing existing skills required to attain personal goals within the social context but also for acquiring new ones (e.g., Vaughn et al., 2016). Additionally, they provide a context for the co-construction of social relationships with a strong impact on an individual’s well-being later in life (Rubin et al., 2009).

In societies where young children are enrolled for several hours a day in child-care centers, the peer group becomes an even more important context. In Portugal, according to the Organization for Economic Co-operation and Development (2019), 92% of children between the ages of 3 and 5 are enrolled in preschool (higher than the average for the OECD, 87%, and the EU, 90%). These preschool experiences increase children’s opportunities to interact with peers as potential play companions and possibly enable them to benefit from these interactions. For some children, such experiences may represent increased challenges, if they lack the skills to initiate and maintain positive exchanges with peers (Coplan et al., 2015). The cumulative effects of these sub-optimal peer experiences may put these children at risk for later psychosocial maladjustment (e.g., Vaughn et al., 2016). Thus, it is important to be able to identify and characterize groups of children with similar social or non-social play behaviors, so early social difficulties can be distinguished and preventive strategies can be implemented to avoid the onset of less healthy developmental trajectories.

Although peer interactions may occur during other group activities, play seems to be a more frequent context where these interactions take place. During the preschool years, play becomes more salient and progressively more socially sophisticated, with the expansion of children’s social network and cognitive and emotional abilities (Coplan and Arbeau, 2009). Social play is grounded in children’s abilities to initiate and engage with peers in a shared activity, using skills such as cooperation, imaginary play, and turn-taking (e.g., Coplan et al., 2015). During these transactions, children participate in social episodes in which their actions both are responses to other’s behaviors and constitute new stimuli that may elicit a response from the partner (Rubin et al., 2006; Coplan et al., 2015). The quality of these playful interactions has an impact on children’s levels of acceptance by their peers and how they develop friendships (e.g., Vaughn et al., 2000).

For several decades, researchers have tried to describe and understand why some children find peer interactions challenging, but the literature has been characterized by inconsistencies. Rubin and colleagues (Rubin and Asendorpf, 1993; Coplan et al., 1994; Coplan and Rubin, 1998a; Rubin, 2001; Rubin et al., 2009) have made major contributions to the field by using a consistent and empirically based taxonomy of non-social behaviors. Non-social play behaviors tend to be described as the consistent display (over time and different contexts) of solitary activity and/or behaviors while in the presence of potential play partners that neither initiate nor maintain a social transaction (e.g., Coplan et al., 2015). A variety of non-social behaviors have been described that may reflect different motivational mechanisms: Reticent behavior includes a cluster of solitary acts such as continuous onlooking toward a potential play partner without attempting to join in or being unoccupied while at a distance from peers. These children seem to want to engage in play with their peers but are anxious and afraid to do so, leading them to avoid interaction. Solitary–passive behaviors involve constructive play and object exploration while playing alone (e.g., playing with building blocks). These children tend not to approach their peers during play, but while seemingly disinterested in engaging with them, they do not avoid/reject them if approached. Solitary–active play behaviors refer to the display of functional play in the form of recurrent sensory–motor activities with or without objects, or solitary dramatic/pretense play, in the presence of peers (e.g., Coplan et al., 1994; Coplan and Rubin, 1998b).

Due to the centrality of play for children’s early development, researchers have tried to understand the environmental and genetic precursors of children’s play quality (e.g., Cheah et al., 2001). Typically, the focus has been on individual characteristics such as age, sex, and temperament and less on contextual and dyadic variables such as parenting beliefs/strategies and parent–child interactions. To disentangle the complexity and diversity of this phenomenon, Rubin and Mills (1991) have proposed a model emphasizing transactions between a child’s individual characteristics and parenting practices as precursors of children’s social/non-social play. For example, a child with an inhibited temperament may react anxiously to new and challenging situations and evoke responses such as excessive control or intrusiveness from parents. These parenting behaviors have been linked to reticence and social withdrawal (Hastings et al., 2010). However, this research has been focused mainly on mothers, while the role of fathers has been understudied across development (Cabrera et al., 2018).

Following Rothbart and Ahadi’s (1994) psychobiological approach, children’s temperament is centered on individual differences in the way they react to the world and how they regulate behaviors and emotions. Since navigating the world requires reacting, regulation, and behaving accordingly, children’s ability to self-regulate and their reactivity to other stimuli affects the quality of their playful transactions with adults and peers (Slot et al., 2017). Studies have shown that children with fearful or anxious temperamental traits are more behaviorally inhibited and tend to engage in non-social play (e.g., Fox and Calkins, 1993), to disengage from peers, and to withdraw from social interactions (Buhs and Ladd, 2001). Moreover, reticent behaviors observed in the context of play have been associated with temperamental shyness and fearfulness (Henderson et al., 2004). The existing literature does not tend to report sex differences in terms of prevalence of social and non-social play behaviors (see Rubin et al., 2009 for a review). Nonetheless, the consequences of non-social behaviors seem to be different for boys vs. girls due to social gender bias; e.g., in a non-systematic review by Doey et al. (2013), several studies suggested that shy, withdrawn behaviors of boys are associated with more negative responses by peers, parents, and teachers.

Typically, parents are children’s first social partners and caregivers, with the quality of parental care and the experiences co-constructed within these relationships being cornerstones for the way children adapt and organize their expectations, behaviors, and emotions in present and future social experiences outside the family (e.g., Sroufe et al., 2005). The literature often describes differences in the ways mothers and fathers interact with their children and suggests that fathers play more than they are involved in care (e.g., Monteiro et al., 2017b) and that their play is more active and physical, in comparison to mothers. Moreover, fathers are described as being more encouraging of their children to explore, take risks, and push limits (e.g., Lamb and Lewis, 2011; Fletcher et al., 2012). In terms of parenting styles and practices, fathers tend to identify themselves as more authoritarian than their spouses (Winsler et al., 2005) and recurring to more authoritarian practices (Russell et al., 2003), especially if they have sons. In Portuguese samples, fathers tend to report being more authoritative than authoritarian (e.g., Pedro et al., 2015; Monteiro et al., 2017b), although when compared to mothers, they report lower levels of the authoritative style (Pedro et al., 2015).

This study is focused on fathers, since there is less information (as in other domains) about their impact on children’s social and non-social play. A few empirical studies have supported the association between the development of children’s shyness and fathers’ parenting behaviors (Hastings et al., 2010). For instance, fathers’ critical and non-supportive parenting styles were associated with teacher reports of elevated anxiety and isolation in preschool-age children (McShane and Hastings, 2009). On the contrary, even when controlling for effects of race, ethnicity, and socioeconomic status, fathers’ sensitive and supportive behaviors are associated with children’s positive outcomes (e.g., Cabrera et al., 2018 for review). Parke (1995) reports that when both mother and father are involved, fathers might be as important as mothers for the development of children’s abilities to positively interact and play with their peers.

### The Current Study

Using Rubin and Mills (1991) model as a framework, the aim of this study was to identify distinct profiles of children with similar patterns of play behaviors using a person-centered approach. This approach does not presume that a single model should fit an entire population or sample; rather, it suggests that multiple, relatively homogeneous subgroups may be found in a given sample or population but that classification categories cannot be determined *a priori* (Howard and Hoffman, 2017). Next, we intended to characterize children’s play profiles (controlling for age) in relation to child’s sex and temperamental characteristics (extroversion, effortful control, and negative affectivity) and father’s parenting styles (e.g., warmth and involvement or corporal punishment) in a developmental period described by researchers (e.g., Lamb and Lewis, 2010) as particularly salient for father–child interactions, since children become more physically, cognitively, emotionally, and socially competent, facilitating the father’s involvement.

## Materials and Methods

### Participants

Two-hundred and forty-three children, their mothers and fathers, as well as their preschool teachers participated in the study. Children were between 36 and 72 months old (*M* = 53.60, *SD* = 11.50), 121 were girls, and 150 had siblings. Father’s age ranged between 24 and 56 years (*M* = 38.08, *SD* = 4.91), with 52% of the fathers having primary to high school education and 48% a university degree; 95% worked full time. Mother’s age ranged between 24 and 47 years (*M* = 36.13, *SD* = 4.37), with 34.5% having primary to high school education and 65.4% a university degree; 90% worked full-time. Families were within the middle-class range according to Portuguese standards. Sixty-two preschool teachers with an average of 40.57 years of age (*SD* = 8.34), all with a university degree in early education, also participated.

### Procedures/Instruments

This study is part of a larger project aiming to study the impact of father’s involvement in children’s socio-emotional development during the first years. Parents and teachers were informed of the main objectives of the project and signed an informed consent prior to any data collection. Mothers completed the sociodemographic and child’s temperament questionnaires; fathers completed the parenting styles questionnaire for the target child participating in the project. Each preschool teacher reported typical play behavior of, on average, four children in their classroom. The classrooms were organized by child’s age, with 15–20 children in the group.

The *Preschool Play Behavior Scale* (Coplan and Rubin, 1998a) is an 18-item questionnaire with five dimensions describing children’s behaviors during free play, in the presence of their peers. It aims to differentiate social play and different types of non-social behaviors (reticent, solitary–passive, solitary–active, and rough). The validated Portuguese version (Monteiro et al., 2017a) maintained, through a confirmatory factor analysis, the five-dimension model, retaining 14 of the original items: Reticent behavior refers to behaviors characteristic of children who observe their peers without participating (e.g., “wanders by the classroom without any purpose”); solitary–passive describes exploratory and constructive behaviors without social engagement (e.g., “plays alone, exploring toys or objects, trying to figure out how they work”); solitary–active describes dramatic solitary play (e.g., “plays make-believe but alone”); social play includes peer playing and active participation in constructive peer interactions (e.g., “talks with other children while playing”); and rough play refers to physical play and play fighting (e.g., “engages in simulated and enjoyable/fun fights with other children”). Preschool teachers answered on a five-point scale (1 – never, 3 – sometimes, 5 – always). Cronbach’s alpha analysis revealed acceptable levels for all dimensions: reticent (α = 0.76), solitary–passive (α = 0.72), solitary–active (α = 0.73), social (α = 0.89), and rough play (α = 0.94).

The *Children’s Behavior Questionnaire* – *Short Form Version* (Putnam and Rothbart, 2006; Franklin et al., unpublished) assesses the child’s temperament as the constitutionally based individual differences in reactivity and self-regulation, influenced over time by heredity and experience (e.g., Rothbart and Ahadi, 1994). In the Portuguese version (Lopes, 2011), 73 items were retained (of the 94 original) and organized in the 15 scales fitting Rothbart’s three-dimension model: extroversion, referring to high activity levels, impulsivity, and sociability (e.g., “likes to slide down or do other adventurous activities”); effortful control, referring to the ability to plan adequate responses/suppress inappropriate responses (e.g., “can wait for new activities when asked to wait”); and negative affectivity, referring to the expression of feelings of fear, sadness, and anger (e.g., “throws tantrums when doesn’t get what he/she wants”). Mothers answered on a seven-point Likert-like scale (1 – “extremely untrue of your child,” 3 – “slightly untrue of your child,” 7 – “extremely true of your child). All dimensions reached acceptable Cronbach’s alpha levels: extroversion (α = 0.82), effortful control (α = 0.82), and negative affectivity (α = 0.73).

The *Parenting Styles and Dimensions Questionnaire* – *Short Version* (Robinson et al., 2001), validated for Portuguese samples by Pedro et al. (2015), maintained the 32 items that can be organized in terms of parenting styles and dimensions. For the purpose of this study, only the dimensions and practices were used: corporal punishment, punitive strategies, and verbal hostility, characterized with high restrictiveness and low responsiveness (e.g., “uses threats as punishment with little or no justification”), and warmth and involvement, reasoning/induction, and democratic participation, associated with high responsiveness and high demandingness (e.g., “explains the consequences of child’s behavior”). Fathers reported on a five-point Likert scale (1 – never, 3 – about half of the time, 5 – always). The Cronbach’s alphas for corporal punishment (0.67), punitive strategies (0.70), warmth and involvement (0.65), reasoning/induction (0.65), and democratic participation (0.70) were all acceptable, with the exception of verbal hostility (0.52), which was not considered for further analysis.

## Play of Analysis

A cluster analysis was conducted to identify children’s play behavior profiles conducted in a two-stage grouping procedure (Hair and Black, 2000). A hierarchical cluster analysis was performed using Euclidean distances for the initial observations, using the Ward method to identify the clusters. Then, a non-hierarchical method of clustering cases (k-means) was used to optimize the subject’s distribution in each cluster. In order to analyze the differences between profiles, considering the play behaviors, a multivariate analysis of variance (MANOVA) was used, and in case of significant effects, a *post-hoc* (Tukey) test. Third, a multivariate analysis of covariance (MANCOVA) was performed to test possible differences in the established play profiles in terms of the child’s temperament and parenting dimensions, considering child’s sex and using age as a covariate. Pillai’s Trace criterion (V) was selected as the multivariate test to assess the statistical significance of the group effect, due to its robustness with unequal sample sizes (Tabachnick and Fidell, 2007). When significant multivariate effects were identified, subsequent univariate analyses of covariance (ANCOVAs) were computed, followed by pairwise comparisons with Bonferroni corrections.

## Results

**FIGURE 1:**
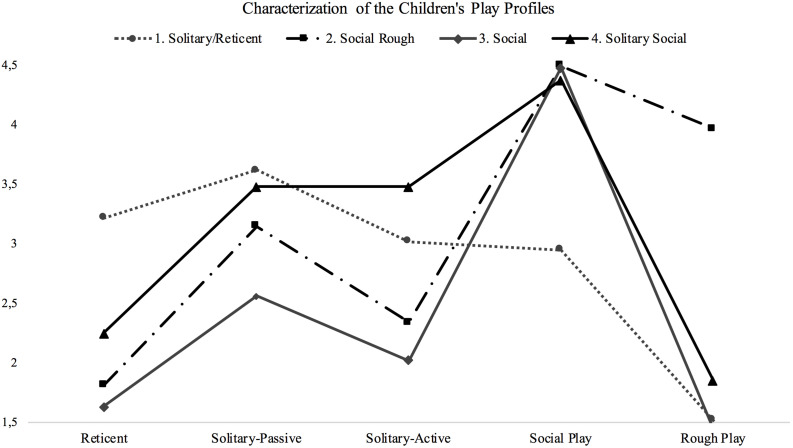
Final four-cluster solution based on children’s play behaviors and children’s play profile characterization. The *x*-axis represents the children’s play behaviors, and the *y*-axis, the averages on a five-point scale. The lines illustrate the averages of play behaviors for each cluster/profile.

In order to identify children with similar play behaviors, a cluster analysis was conducted, with a hierarchical cluster analysis using Euclidian distances and a parsimony assessment of the agglomeration coefficients and the dendrogram, revealing a four-cluster solution (*R*^2^ = 51.05%), followed by a k-means cluster analysis, to enhance subject’s distribution, with the final four-cluster solution (*R*^2^ = 53.18%): Solitary/Reticent (*n* = 33, 13.69% of the sample), Social Rough (*n* = 77, 31.95%), Social (*n* = 60, 24.90%), and Social Solitary (*n* = 71, 29.46%). Figure 1 shows the means of play behaviors for each play profile.

To better understand the play profiles, differences between the four profiles regarding the five categories of play behaviors were analyzed with a MANOVA and *post-hoc* tests (Tukey); a significant multivariate effect [*V* = 1.70, *F*(15, 711) = 61.92, *p* < 0.00, π = 0.57] and consequent significant univariate effects for all play behaviors were found. The results are presented in Table 1. These results confirm that the constituted groups include children with statistically different profiles regarding the dimensions of social and non-social play behavior. The Solitary/Reticent profile has significantly lower scores of social play and significantly higher scores of reticent behaviors than the remaining three profiles. The three social profiles do not show significant differences between them in terms of social play, but we could identify significant differences in specific types of behaviors. For example, the Social Rough profile shows significantly higher scores of rough play, and the Social Solitary profile displays significantly higher scores of solitary–passive and solitary–active behaviors.

**TABLE 1:** Comparison of children’s play behavior dimensions between play profiles.

**PPBS**	**1. Solitary/Reticent (*n* = 33)**	**2. Social Rough (*n* = 77)**	**3. Social (*n* = 60)**	**4. Social Solitary (*n* = 73)**	**ANOVAs**			**Tests *aposteriori***
	
	**M (SD)**	**M (SD)**	**M (SD)**	**M (SD)**	**F**	**p**	**η^2^*_*p*_***	
Reticent	3.22 (0.69)	1.81 (0.55)	1.63 (0.43)	2.25 (0.52)	72.15*	0.00	0.48	1 > 2***, 1 > 3***, 1 > 4***, 2 < 4***, 3 < 4***
Solitary–passive	3.62 (0.65)	3.15 (0.68)	2.56 (0.54)	3.48 (0.61)	31.08*	0.00	0.28	1 > 2***, 1 > 3***, 2 > 3***, 2 < 4**, 3 < 4***
Solitary–active	3.02 (0.77)	2.34 (0.93)	2.02 (0.52)	3.48 (0.70)	50.35*	0.00	0.39	1 > 2***, 1 > 3***, 1 < 4*, 4 > 2***, 4 > 3***
Social play	2.95 (0.58)	4.50 (0.49)	4.48 (0.43)	4.38 (0.40)	101.16*	0.00	0.56	1 < 2***, 1 < 3***, 1 < 4***
Rough play	1.52 (0.68)	3.97 (0.84)	1.45 (0.54)	1.85 (0.76)	181.80*	0.00	0.70	1 < 2***, 2 > 3***, 2 > 4***, 3 < 4**

*^∗^p < 0.05, ^∗∗^p < 0.01, ^∗∗∗^p < 0.001.*

A MANCOVA was used to assess differences in play profiles in terms of the child’s temperament and father’s parenting dimensions, considering children’s sex and using age as a covariable. After controlling for children’s age, a significant multivariate effect was found between the play profiles, the dimensions of children’s temperament, and the fathers’ parenting dimensions [*V* = 0.18, *F*(24, 681) = 1.81, *p* = 0.01, π = 0.99]. The results are presented in Table 2. Regarding *children’s temperament*, a univariate effect between the play profiles and effortful control [*F*(3, 232) = 4.48, *p* = 0.004, $\eta_{p}^{2}$ = 0.06] was found. Pairwise comparisons with Bonferroni corrections showed that children with Solitary/Reticent and Social Rough profiles had significantly lower scores on effortful control when compared to children with a Social profile, while for *father’s parenting*, a significant univariate effect was found between play profiles and father’s punitive strategies [*F*(3, 232) = 4.66, *p* = 0.003,$\eta_{p}^{2}$ = 0.06]. Pairwise comparisons with Bonferroni corrections showed that children in the Social Rough profile have fathers whose parenting is characterized by statistically significant higher scores in the punitive strategies when compared with children in the Social and Social Solitary profile. Additionally, children in the Solitary/Reticent profile have fathers who report significantly less punitive strategies when compared with children in the Social Rough profile.

**TABLE 2:** Comparison of children’s temperament dimensions and fathers’ parenting styles between play profiles.

**PPBS**	**1. Solitary/Reticent (*n* = 33)**	**2. Social Rough (*n* = 77)**	**3. Social(*n* = 60)**	**4. Social Solitary (*n* = 73)**	**ANOVAs**			**Tests *aposteriori***
	
	**M (SD)**	**M (SD)**	**M (SD)**	**M (SD)**	**F**	**p**	**η^2^*_*p*_***	
**Children’s temperament**								
Extroversion	4.91 (0.15)	5.23 (0.15)	4.83 (0.13)	4.77 (0.10)	2.19	0.09	0.03	
Effortful control	5.34 (0.10)	5.34 (0.10)	5.69 (0.09)	5.64 (0.07)	4.48*	0.00	0.06	1 < 3*, 2 < 3*
Negative affectivity	4.64 (0.10)	4.49 (0.10)	4.40 (0.09)	4.59 (0.07)	1.42	0.24	0.02	
**Fathers’ parenting domains**								
Warmth and involvement	4.14 (0.09)	4.25 (0.09)	4.17 (0.08)	4.30 (0.06)	1.02	0.38	0.01	
Reasoning/Induction	3.75 (0.10)	3.72 (0.10)	3.55 (0.09)	3.69 (0.07)	1.00	0.39	0.01	
Democratic participation	3.68 (0.12)	3.67 (0.12)	3.68 (0.10)	3.65 (0.08)	0.03	0.99	0.00	
Corporal punishment	1.61 (0.09)	1.81 (0.09)	1.56 (0.08)	1.54 (0.06)	2.38	0.07	0.03	
Punitive strategies	1.45 (0.10)	1.82 (0.10)	1.48 (0.08)	1.38 (0.07)	4.66*	0.00	0.06	1 < 2*, 2 > 3*, 2 > 4**

*^∗^p < 0.05, ^∗∗^p < 0.01.*

No significant multivariate was found for sex [*V* = 0.02, *F*(8, 225) = 0.59, *p* = 0.78, π = 0.27], but a significant multivariate interaction was revealed between play profiles and sex [*V* = 0.19, *F*(24, 681) = 1.87, *p* = 0.01, π = 0.99]. For parenting, a significant result for father’s punitive strategies [*F*(3, 241) = 3.84, *p* = 0.01, $\eta_{p}^{2}$ = 0.05], was found; scores were higher for boys especially if they had a Solitary/Reticent play profile (*M* = 1.60, *SD* = 0.12) and for girls with a Social Rough play profile (*M* = 2.08, *SD* = 0.18).

## Discussion

Based on teachers’ descriptions of children’s play behaviors, in the school context, four profiles were identified: Solitary/Reticent, Social Rough, Social, and Social Solitary. The Solitary/Reticent profile is described as a non-social profile since it has the lowest scores of social play, and it is also defined by higher scores of reticent behaviors and moderate scores of solitary behaviors. The Social, Social Rough, and Social Solitary were considered social profiles, since no significant differences were found for social play, although differences were found for rough play and solitary–passive behaviors, highlighting that, at least in this sample, children characterized as social are not a simple and homogenous group.

As expected, children who usually engaged in social play were described as having higher levels of effortful control compared to children who displayed more frequent non-social behaviors. Effortful control entails the capacity to direct attention and activate or disactivate behavioral responses in order to adapt to the situation (Putnam and Rothbart, 2006) and is thus associated with higher social competence. In addition, children with a Social Rough profile scored significantly lower on effortful control than children with a social play profile. This result was not expected, as for example, in Peterson and Flanders (2005) model, it is argued that rough-and-tumble play is a key contributor to the development of self-regulation. More studies are necessary to understand if this more “disorderly” type of play is in fact associated with children’s lower regulatory abilities or, since it is more challenging, it is perceived less positively by adults.

Considering father’s parenting styles, our findings showed significantly higher scores of father’s punitive strategies in children with a Social Rough profile. This type of strategy is characterized by the disciplinary use of punishments without accompanying explanations or reasons for doing so (Robinson et al., 2001), although we should interpret these results with caution since on average, these values are relatively low. Despite studies describing fathers as encouraging of this type of active, physical, and “rough” play, in our sample, it is possible that fathers perceive this type of behavior (play fights, rough, and tumble) as more challenging to family and group norms, since it can be perceived by adults as a form of aggression and an unsafe activity (Panksepp, 1993), and therefore use more punitive strategies (although the average values are low). Future studies should explore possible cultural differences in the way parents and teachers perceive this type of play. In addition, as recent studies (e.g., Scarzello et al., 2016) suggest that parenting and educational practices are greatly influenced by parents’ knowledge of child development, future research should also consider how fathers’ knowledge of child development and expected behaviors in each developmental stage may influence the parenting practices adopted.

Although a sex effect was not found, a significant interaction effect between play profiles and child’s sex emerged regarding father’s use of punitive strategies. Fathers reported more frequent use of this parenting practice if they had sons with a Solitary/Reticent profile and if they had daughters with a Social Rough profile. These results are particularly interesting considering the existing literature regarding the possible influence of gender stereotypes and cultural norms in the sex differences found for the consequences of non-social behaviors (see Rubin et al., 2009). In Western European cultures (specially in southern countries), stereotypical gender norms suggest that males should be socialized to be assertive and dominant, and females are expected to be softer and nurturing (e.g., Gebauer et al., 2013). Disruption of social expectations and norms of how boys and girls should behave tends to lead to more negative responses from parents, teachers, and peers (Rubin et al., 2009; Doey et al., 2013). Interestingly, Lytton and Romney (1991) found that this gender bias seems to be more salient in fathers than mothers. A qualitative study assessing how parents think about fathers’ rough-and-tumble play (StGeorge et al., 2018) found that although fathers believe this type of play should occur equally with girls and boys, in reality, it does not, with some justifying that girls are more delicate and, as such, they should play more gender-appropriate games. Alternatively, some studies (Jacklin et al., 1984) suggest that girls incite less of this type of play from their fathers.

### Limitations and Future Research

Some limitations can be identified, namely, that this is not a longitudinal study and that it relies on self-reports. Future studies should also include observational measures, such as the *Play Observation Scale* (Rubin, 2001), in order to provide a more refined taxonomy of children’s play behaviors and their motivations. Additionally, even though the aim of the study was to explore fathers, future studies should also include mothers, which would allow testing for main and interaction effects of both caregivers (e.g., Cabrera et al., 2018).

In this sample, we did not find strict categories of children’s play behaviors; instead, and according to their teachers, children seem to resort to different types of behaviors during their peer interactions, as multiple modes of adaptation within the peer group. Further studies should consider a person-centered approach, in order to attain more detailed knowledge of how play profiles emerge and to understand its predictors, correlates, and outcomes (Howard and Hoffman, 2017), although based on self-reports, different and independent sources were used, therefore increasing the study validity. Another innovative aspect is the focus on the father’s role in the child’s social and non-social behaviors, since the literature is mostly focused on mothers (e.g., McShane and Hastings, 2009; Hastings et al., 2010), and as Cabrera et al. (2018) stated, fathers are parents too and should be fully integrated both in research and in parenting interventions. Since children who consistently display a low quality of peer interactions may be more susceptible to later social–emotional difficulties (Cheah et al., 2001), having the means to identify these difficulties early on should be a priority in early education.

## Data Availability Statement

The raw data supporting the conclusions of this article will be made available by the authors, without undue reservation, to any qualified researcher.

## Ethics Statement

This study was carried out in accordance with the recommendations of the American Psychological Association Ethical Guidelines and was approved by Iscte-Instituto Universitário de Lisboa’s Ethics Committee. Participants provided their written informed consent to participate in the study in accordance to the Declaration of Helsinki.

## Author Contributions

LM and CS: conception of the work and acquisition of data. CS and OR: data analysis. LM, CS, OR, and BV: interpretation of data, drafting the manuscript, final approval of the version to be published, and agreement to be accountable for all aspects of the work. All authors contributed to the article and approved the submitted version.

## Conflict of Interest

The authors declare that the research was conducted in the absence of any commercial or financial relationships that could be construed as a potential conflict of interest.
